# Standardized Clinical Assessment and Management Plan Enhances Neonatal Outcomes in Prenatally Diagnosed Congenital Heart Disease

**DOI:** 10.1007/s00246-025-03923-4

**Published:** 2025-06-14

**Authors:** Lior Kashani Ligumsky, Angela Desmond, Vanessa Kirschner, Guadalupe Martinez, Joanne Newens, Gary Satou, Kara Calkins, Yalda Afshar

**Affiliations:** 1 Division of Maternal Fetal Medicine, Department of Obstetrics and Gynecology, David Geffen School of Medicine, University of California Los Angeles, Los Angeles, CA USA; 2 School of Medicine, Tel Aviv University, Tel Aviv, Israel; 3 Division of Neonatology & Developmental Biology, Neonatal Research Center of the Children’s Discovery and Innovation Institute, Department of Pediatrics, David Geffen School of Medicine, University of California Los Angeles, Los Angeles, CA USA; 4 Division of Pediatric Cardiology, Department of Pediatrics David Geffen School of Medicine, UCLA Mattel Children’s Hospital, Los Angeles, CA USA; 5 Molecular Biology Institute, University of California, Los Angeles, USA

**Keywords:** Congenital heart disease, Neonatal outcomes, Standardized Clinical Assessment and Management Plan (SCAMP), Preterm birth, Cesarean birth

## Abstract

Congenital heart disease (CHD) remains a leading cause of neonatal morbidity and mortality. The University of California Fetal Consortium implemented a Standardized Clinical Assessment and Management Plan (SCAMP) to optimize birth timing and mode of birth in pregnancies complicated by fetal CHD. This study evaluates the impact of SCAMP implementation on neonatal outcomes, specifically survival to hospital discharge, birth weight, and hospital length of stay. A retrospective cohort study was conducted comparing neonates with prenatally diagnosed CHD before (historical cohort) and after (intervention cohort) SCAMP implementation. Neonatal data, including mode of birth, birth weight, survival to discharge, and CHD classification was collected from five UC medical centers. Comparative analyses were performed using t-tests for continuous variables and chi-square tests for categorical variables. A total of 414 neonates met inclusion criteria (167 in the historical cohort, 247 in the intervention cohort). The overall neonatal survival rate to discharge was significantly higher in the intervention cohort (91.1% vs. 83.1%, p = 0.04). Survival following cesarean birth was also improved in the intervention cohort (89.1% vs. 78.1%, p = 0.04), while no significant differences were found for induction of labor or spontaneous vaginal births. Birth weight was significantly higher post-SCAMP (2977 g vs. 2838 g, p = 0.01), and hospital length of stay was significantly shorter (11.5 vs. 26 days, p < 0.01). Survival differences by CHD risk classification were not statistically significant. SCAMP implementation was associated with improved neonatal survival, increased birth weight, and reduced hospitalization duration in CHD-affected pregnancies. These findings underscore the importance of standardized perinatal management in optimizing outcomes for neonates with CHD.

**Supplementary Information:**

The online version contains supplementary material available at 10.1007/s00246-025-03923-4.

## Introduction

Congenital heart disease (CHD) remains one of the most prevalent congenital anomalies, affecting approximately 1% of live births globally, and is a major contributor to infant morbidity and mortality [1, 2]. Despite significant advancements in prenatal screening and postnatal medical and surgical interventions, managing pregnancies complicated by fetal CHD continue to present unique challenges [3–5].

While a prenatal diagnosis of CHD facilitates early coordination of neonatal care, it has also been associated with unintended consequences, including increased rates of early-term and preterm delivery and cesarean births [6–8]—both of which are linked to poor neonatal outcomes, including respiratory distress syndrome, transient tachypnea of the newborn, feeding difficulties, hyperbilirubinemia, hypoglycemia, and hypothermia. These complications lead to increased ICU admissions, prolonged hospital stays, and higher healthcare costs, with potential long-term neurodevelopmental implications even for late preterm neonates. Given these risks, evaluating neonatal outcomes such as survival to discharge and hospital length of stay is critical to assessing the impact of standardized perinatal management strategies like SCAMP [8–12].

**Fig. 1:**
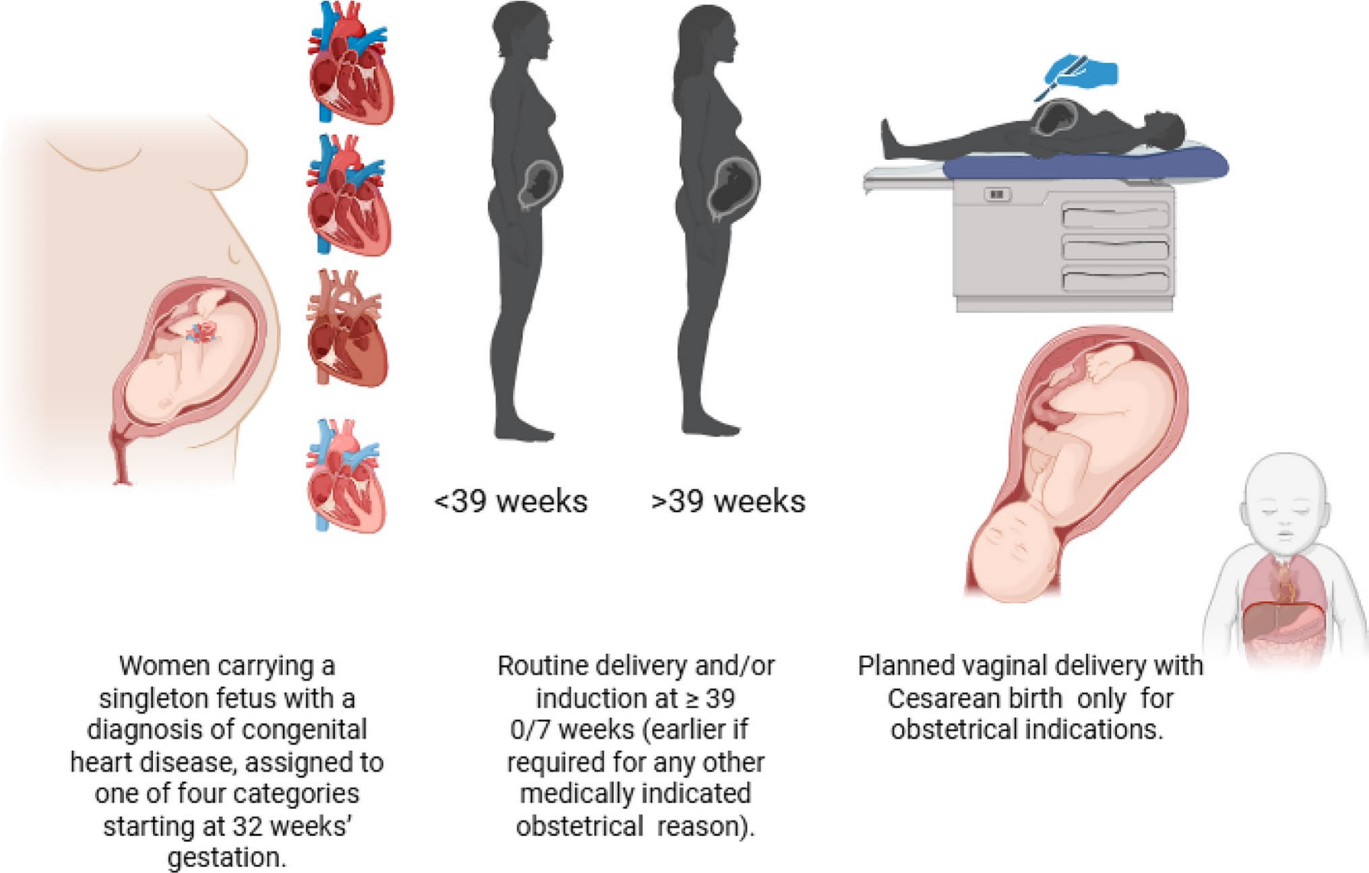
SCAMP Guidelines for Timing and Mode of Delivery in Pregnancies Complicated by Fetal CHD. The SCAMP (Standardized Clinical Assessment and Management Plan) recommends routine delivery or induction at ≥ 39 + 0 weeks’ gestation, unless earlier delivery is indicated for obstetric or medical reasons. Vaginal delivery is preferred, with cesarean delivery (CD) reserved for obstetric indications. Exceptions include consideration of CD for fetuses with complete heart block or for CHD lesions requiring coordinated delivery due to anticipated hemodynamic instability at birth. Women carrying a singleton fetus with a diagnosis of CHD, categorized into one of four lesion-based risk groups beginning at 32 weeks’ gestation, were included in the intervention cohort if delivery was planned at one of five UCfC sites between May 2018 and December 2019. A historical control cohort was drawn from a prior study conducted from 2011 to 2013

In response to these challenges, the University of California Fetal Consortium (UCfC) implemented a Standardized Clinical Assessment and Management Plan (SCAMP) to improve maternal and neonatal outcomes in pregnancies affected by CHD (Fig. 1). The SCAMP standardized decision-making around the timing and mode of delivery, prioritizing delivery at ≥ 39 weeks to reduce unnecessary early-term births and promote vaginal birth over elective cesarean. Our previous study demonstrated a 11% reduction in non-medically indicated cesarean births and a 15% decrease in early-term deliveries. However, its impact on neonatal survival remains incompletely understood [13]. In fetal CHD cases, a SCAMP-driven approach aims to mitigate iatrogenic premature birth while maintaining neonatal stability, thereby improving birth weight and survival outcomes.

Building on this success, the present study evaluated the impact of the SCAMP on neonatal outcomes, focusing on survival to discharge, birth weight, and hospital length of stay. By comparing neonatal data from historical cohorts and those managed under the SCAMP, we seek to investigate how standardized management of CHD, that have already been shown to improve maternal outcomes, influences neonatal outcomes in this vulnerable population.

## Methods

A multi‐institutional retrospective study review board reliance registry provided approval for the study (IRB #10‐04093) and informed consent was waived. Neonatal data were collected and analyzed from historical (pre-SCAMP) and intervention (post-SCAMP implementation) cohorts. The historical cohort consisted of neonates delivered between January 2016 and December 2016, and the intervention cohort comprised neonates born between May 2018 and December 2019. Neonates with complete data on birth mode, birth weight, and survival at discharge were included for analysis.

### Study Population

The intervention cohort included neonates whose mothers were part of the multi-institutional UCfC SCAMP program. The UCfC comprises the five University of California campuses affiliated with university medical centers that offer prenatal diagnosis and treatment (UCfC: UC Davis, UC Irvine, UC Los Angeles, UC San Diego, and UC San Francisco). The UCfC was established to better study pregnancies affected with maternal and fetal diseases, including CHD and to define treatment practices within our health system. This multi‐institution consortium developed a SCAMP for pregnant women with a prenatal diagnosis of fetal CHD, based on current practices and best available evidence. The SCAMP guidelines included recommendations for delivery timing and mode, specifically routine delivery at ≥ 39 weeks and planned vaginal birth unless cesarean birth as indicated for obstetrical or fetal reasons, such as complete heart block or anticipated hemodynamic instability. Neonates were then further categorized by their fetal CHD lesions. The CHD lesions were categorized based on predicted risk of hemodynamic instability in the delivery room or first days of life, based on the American Heart Association guidelines [14]. This included category 1, CHD without predicted risk of hemodynamic instability in the delivery room or first days of life (ventricular septal defects, atrioventricular septal defects); category 2, CHD with minimal risk of hemodynamic instability in the delivery room but requires postnatal catheterization/surgery (ductal-dependent lesions); category 3, CHD with likely hemodynamic instability in the delivery room requiring immediate specialty care for stabilization (D-transposition of the great arteries); category 4, CHD with expected hemodynamic instability with placental separation requiring immediate catheterization/surgery to improve survival (hypoplastic left heart syndrome or D-transposition of the great arteries with restrictive of intact atrial septum), unknown/other cardiac lesion (Tables 1, 2, 3).

**Table 1:** Demographic data and neonatal outcomes

	Historical (n = 167)	Intervention (n = 247)	P value
Maternal age (years)	30.7	33.1	< 0.01
Gravida (median)	2	2	0.12
Parity (median)	1	1	0.94
Gestational age at delivery < 39 weeks n(%)	113 (67.7)	141 (57.1)	0.03
Neonatal birth weight (g)	2838 ± 641	2977 ± 709	0.04
Cesarean birth < 39 weeks’ gestation	62/113 (54.9)	71/141 (50.4)	0.51

Cardiac classification	Historical (n = 167)	Intervention (n = 216)	P value
Category 1 cardiac lesion	67 (40.1%)	44 (20.4%)	0.029
Category 2 cardiac lesion	60 (36.9%)	110 (50.9%)	< 0.01
Category 3 cardiac lesion	19 (9.6%)	35 (16.2%)	0.03
Category 4 cardiac lesion	18 (10.8%)	22 (10.2%)	0.53
Other/unknown	3 (1.8%)	5 (2.3%)	0.48

Values are *n(*%) or median (Q1–Q3), unless otherwise indicated

^a^Percentages displayed under survival at discharge—spontaneous birth represent (*n*) of pregnancies that reported a spontaneous vaginal delivery, not of total *n*

^b^Percentages displayed under survival at discharge—IOL represent (*n*) of pregnancies that reported an IOL, not of total *n*

^c^Percentages displayed under survival at discharge—cesarean birth represent (*n*) of pregnancies that reported a cesarean birth, not of total *n*

**Table 2:** Characterization and neonatal outcomes by fetal CHD

	Historical (n = 167)	Intervention (n = 247)	P value
Survival at discharge n(%)	147 (83.1)	225 (91.1)	0.01
Survival at discharge—spontaneous vaginal birth n, total (%)	40/45 (88.8)	81/90 (90.0)	0.81
Survival at discharge—IOL n, total (%)	36/40 (90.0)	54/56 (96.4)	0.11
Survival at discharge—caesarean birth n, total (%)	64/82 (78.0)	90/101 (89.1)	0.04
Length of stay (days)	26 (14–60)	11.5 (3–40.8)	< 0.01

**Table 3:** Characterization and neonatal outcomes by fetal CHD classifications

	Birth weight (g)	Survived to discharge n(%)	Cesarean birth n(%)	Gestational age < 39 weeks n(%)	Cesarean birth before 39 weeks n(%)
Category 1 cardiac classification					
Historical n = 67	2942.4	56(83.5)	33(49.2)	41(61.2)	23(34.3)
Intervention n = 44	2886.9	42(95.5)	19(43.2)	22(50)	13(29.5)
P value	0.63	0.07	0.54	0.27	0.67
Category 2 cardiac classification					
Historical n = 60	2699.8	48(80)	27(45)	45(75)	23(38.3)
Intervention n = 110	2989.6	103(93.6)	46(41.8)	61(55.4)	35(31.8)
P value	0.01	< 0.01	0.3	0.007	0.42
Category 3 cardiac classification					
Historical n = 19	3237.7	18(94.7)	7(36.8)	9(47.4)	4(21.1)
Intervention n = 35	3080.314	29(82.8)	18(51.4)	19(54.3)	10(28.6)
P value	0.38	0.21	0.33	0.65	0.52
Category 4 cardiac classification					
Historical n = 18	2655.9	16(88.9)	12(66.7)	16(88.9)	10(55.6)
Intervention n = 22	2874.1	16(72.7)	10(45.4)	14(63.6)	8(36.4)
P value	0.24	0.19	0.19	0.04	0.27

### Data Collection

Neonatal data, including mode of birth (vaginal birth, induction of labor (IOL), and cesarean birth), birth weight, survival to discharge and cardiac classification were collected via retrospective chart review at each of the participating sites.

### Outcome Measures

The primary outcome was survival to discharge. Secondary outcomes included birth weight and survival differences based on birth mode and cardiac classification. Additional data on site-specific standard management practices were gathered from maternal–fetal medicine physicians and pediatric cardiologists to maintain site confidentiality.

### Statistical Analysis

Outcomes were compared between the intervention cohort (post-SCAMP) and historical control cohort. Comparative statistical analyses were performed using t-tests (or Wilcoxon rank sum test) for continuous variables (e.g., birth weight) and Chi-square tests (or Fisher’s exact tests) for categorical variables (e.g., birth mode, survival). Analyses were conducted using de-identified data, and results were stored in a Health Insurance Portability and Accountability Act-compliant Research Electronic Data Capture (REDCap) database.

## Results

A total of 414 neonates met the inclusion criteria, with 167 in the historical cohort and 247 in the intervention cohort. The overall survival rate to discharge was higher in the intervention cohort (91.1%) compared to the historical cohort (83.1%, p = 0.04). In the cesarean birth group, survival rates were higher in the intervention cohort (89.1%) compared to the historical cohort (78.1%, p = 0.04). There were no significant differences in survival rates for IOL and spontaneous births between the historical and intervention cohorts (p = 0.8 and p = 0.1, respectively).

The demographic and neonatal outcomes of the intervention (SCAMP) and historical cohorts were compared. Maternal age was higher in the intervention cohort (33 years) compared to the historical cohort (31 years) (p = 0.05). There were no significant differences between the cohorts in terms of gravida and parity, with both cohorts having a median gravida of 2 and median parity of 1 (p = 0.12 and p = 0.90, respectively). Neonatal birth weight was significantly higher in the intervention cohort (2977 ± 709.3 g) compared to the historical cohort (2838 ± 641.7 g) (p = 0.01). The average length of stay was significantly shorter in the intervention cohort (11.5 days) compared to the historical cohort (26 days) (p < 0.01).

For the sub analysis of survival by cardiac classification, only 216 neonates had complete cardiac classification data in the intervention cohort and 167 in the historical cohort. Regarding cardiac classification, a smaller proportion of patients in the intervention cohort were classified as cardiac category 1 (20.4%) compared to the historical cohort (40.1%, p = 0.03). Conversely, a higher proportion of patients in the intervention cohort were classified as cardiac category 2 (50.9% vs. 36.9%, p < 0.01) and cardiac category 3 (16.2% vs. 9.6%, p = 0.03) compared to the historical cohort. There were no significant differences in the proportion of patients in categories 4 or Unknown lesions between the two cohorts (p = 0.53, p = 0.48, respectively).

Further analysis by cardiac category demonstrated the following Table 3:

- In Category 1, survival to discharge was higher in the intervention cohort (95.5%) compared to the historical cohort (83.5%), with a trend toward statistical significance (p = 0.07). There were no significant differences in mean birth weight (2886.9 g vs. 2942.4 g, p = 0.63), cesarean delivery rate (43.2% vs. 49.2%, p = 0.54), or proportion of neonates born at < 39 weeks gestation (50% vs. 61.2%, p = 0.27).
- In Category 2, neonates in the intervention cohort had significantly higher birth weights (2989.6 g vs. 2699.8 g, p = 0.01), higher survival rates (93.6% vs. 80%, p = 0.006), and a lower proportion of births < 39 weeks (55.5% vs. 75%, p = 0.007). Cesarean rates were not significantly different between cohorts (41.8% vs. 45%, p = 0.3).
- In Category 3, no significant differences were found between cohorts in birth weight (3080.3 g vs. 3237.7 g, p = 0.38), survival (82.8% vs. 94.7%, p = 0.21), cesarean rate (51.4% vs. 36.8%, p = 0.33), or proportion of neonates born < 39 weeks (54.3% vs. 47.4%, p = 0.65).
- In Category 4, while birth weight was higher in the intervention cohort (2874.1 g vs. 2655.9 g, p = 0.24), and cesarean delivery was less frequent (45.4% vs. 66.7%, p = 0.19), these differences were not statistically significant. However, the proportion of neonates born at < 39 weeks gestation was significantly lower in the intervention cohort (63.6% vs. 88.9%, p = 0.04). Survival to discharge was not significantly different (72.7% vs. 88.9%, p = 0.19).

## Discussion

Our study found that the implementation of a fetal CHD standardized clinical assessment and management plan was associated with a significant improvement in neonatal survival when compared to the historical cohort. Specifically, the overall survival rate was significantly higher in the SCAMP cohort compared to the historical cohort.

This finding underscores the importance of a structured, evidence-based perinatal management approach that can improve neonatal survival outcomes, particularly in cases of fetal CHD, while also optimizing maternal care and outcomes.

Additionally, we observed that the SCAMP was associated with a significant increase in birth weight and shorter neonatal hospital stays, suggesting that this structured approach not only improved survival but also optimized other neonatal outcomes.

The difference in survival between the two cohorts supports the notion that delaying delivery to ≥ 39 weeks in the absence of obstetric or fetal indications does not negatively impact survival. In fact, this approach may offer protective benefits for neonates with CHD, as evidenced by the improved survival in the SCAMP cohort [9]. Beyond survival, later gestational age at birth is associated with increased maturity, leading to a lower likelihood of NICU admission and reduced risk of respiratory and metabolic complications. Infants born at ≥ 39 weeks are less likely to require respiratory support, intravenous fluids, or prolonged hospitalization, which not only improves immediate neonatal health but also reduces healthcare costs and long-term developmental risks. Additionally, avoiding unnecessary early-term birth promotes earlier parental bonding and facilitates breastfeeding initiation, contributing to improved neurodevelopmental outcomes [15]. Furthermore, while our study found a significant improvement in survival after cesarean birth in the SCAMP cohort compared to the historical cohort, there were no significant differences in survival based on other modes of delivery—including spontaneous vaginal delivery or IOL. This aligns with previous studies indicating that, in the absence of medical indications, planned vaginal birth is a safe option for most fetuses with CHD [7, 16]. Historically, there has been a tendency to schedule cesarean births for coordination of care needs due to concerns about perinatal hemodynamic instability in CHD-affected pregnancies [16–18]. However, our findings suggest that while cesarean birth may be associated with improved survival in some cases, routine cesarean birth does not necessarily confer a universal survival advantage and may instead lead to prolonged neonatal hospitalizations without improving overall outcomes. Therefore, consideration of mode of delivery for previous pregnancies is critical. The SCAMP's emphasis on avoiding early-term elective deliveries and promoting routine delivery at ≥ 39 weeks’ gestation, unless medically indicated, likely contributed to the increased birth weights observed in the intervention cohort [2977 ± 709.3 g Vs (2838 ± 641.7 g) 0.04] [13]. Longer gestation periods associated with SCAMP implementation may have provided neonates with more time to thrive, resulting in higher birth weights. This improves surgical outcomes. Additionally, the significant reduction in neonatal hospital length of stay in the SCAMP cohort (11.5 days vs. 26 days, p < 0.01) highlights improved neonatal stability, as well as potential improvements in care delivery efficiency and reduced healthcare burden [9, 19]. Shorter hospitalizations are associated with fewer complications and a quicker return to normalcy for families, further supporting the benefits of a structured clinical approach. This finding underscores the importance of a structured, evidence-based perinatal management approach. While cesarean delivery rates prior to 39 weeks did not differ significantly between cohorts, the most notable differences in delivery mode were observed among births occurring at or beyond 39 weeks, suggesting a potential influence of SCAMP on term delivery planning. This approach can improve neonatal survival outcomes, particularly in cases of fetal CHD, while also optimizing maternal care and outcomes. Sub-analysis by cardiac classification further supported the benefit of SCAMP. In neonates with Category 1and 2 lesions, survival improved (95.5% vs. 83.5%, p = 0.07), while birth weight increased significantly in Category 2 (2989.6 g vs. 2699.8 g, p = 0.01). Moreover, a lower proportion of births occurred before 39 weeks in the SCAMP cohort for both Category 1, 2 and Category 4 lesions, (p = 0.04), respectively and not only improves general outcomes but may also promote consistency and optimization across lesion severity groups. The unexpectedly low survival rate in Category 1 and 2 neonates in the historical cohort may be partially attributed to higher rates of delivery before 39 weeks and potentially unrecognized genetic or extracardiac anomalies, as full diagnostic evaluations were not universally performed. These findings underscore the importance of standardized delivery planning and coordinated postnatal care, as implemented through the SCAMP pathway. Although these trends were mostly favorable, Category 3 neonates in the SCAMP cohort had lower survival (82.8% vs. 94.7%). While this difference did not reach statistical significance, it may coincided with lower average birth weight, higher cesarean rate, and more frequent delivery before 39 weeks in the SCAMP cohort. These factors may have impacted neonatal resilience and survival despite structured care, highlighting that even within a standardized approach, gestational maturity and birth weight remain critical modifiers of outcome.

Despite these promising results our study has several limitations. First, as a retrospective analysis, it is subject to inherent biases related to data collection and unmeasured confounding factors. Second, although the SCAMP cohorts were broadly comparable, differences in CHD severity between cohorts could have influenced neonatal outcomes independent of the intervention.

This study is limited by its retrospective design and multicenter setting, which may introduce variability in practice patterns and care delivery. The modest sample size may have reduced the power to detect differences in secondary outcomes. Additionally, unmeasured confounders such as socioeconomic factors, undiagnosed genetic syndromes, palliative care decisions, and inconsistencies in diagnostic intensity may have influenced neonatal outcomes. These factors were not uniformly assessed across cohorts, limiting our ability to fully account for their impact.

## Conclusion

The implementation of a fetal CHD SCAMP in a multi-institutional cohort was associated with improved neonatal survival, higher birth weight, and reduced length of hospital stay. These findings suggest that an evidence-based, standardized approach to perinatal CHD management can improve outcomes for neonates affected by CHD. Future studies warrant further investigation with a larger sample size and should focus on long-term outcomes to assess the broader impact of SCAMP on neonatal health beyond the perinatal period.

## Supplementary Information

Below is the link to the electronic supplementary material.

Supplementary file1 (DOCX 150 KB)

## Data Availability

No datasets were generated or analysed during the current study.
