# Supplementary material for: Standardized Clinical Assessment and Management Plan Enhances Neonatal Outcomes in Prenatally Diagnosed Congenital Heart Disease
Source: Pediatr Cardiol. 2025 Jun 14;47(4):1483–9. doi: 10.1007/s00246-025-03923-4 (PMC12945978; doi:10.1007/s00246-025-03923-4)
Supplement: Supplementary file 1 — Supplementary file1 (DOCX 150 KB) [file 246_2025_3923_MOESM1_ESM.docx]

Supplementary

Figure 1:


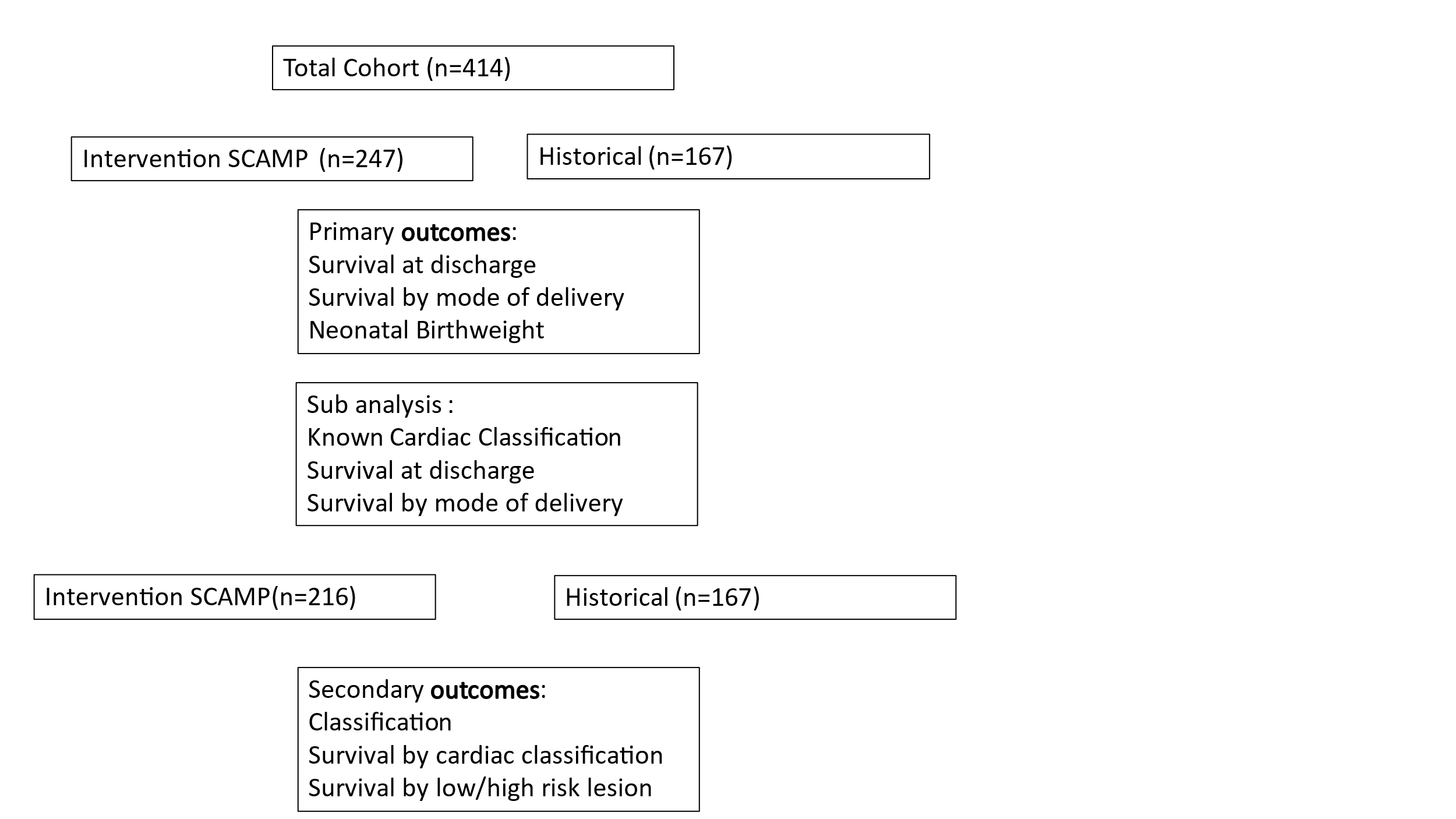


Table 3: Cardiac Classification

| Category | Description | Examples |
| --- | --- | --- |
| Category 1 | CHD without predicted risk of hemodynamic instability in the delivery room or first days of life | Ventricular septal defects, atrioventricular septal defects |
| Category 2 | CHD with minimal risk of hemodynamic instability in the delivery room but requires postnatal catheterization/surgery | Ductal-dependent lesions |
| Category 3 | CHD with likely hemodynamic instability in the delivery room requiring immediate specialty care for stabilization | D-transposition of the great arteries |
| Category 4 | CHD with expected hemodynamic instability with placental separation, requiring immediate catheterization/surgery to improve survival | Hypoplastic left heart syndrome, D-transposition of the great arteries with restrictive or intact atrial septum |
| Unknown/other | CHD cases that do not fit into the defined categories | Other unspecified cardiac lesions |
